# Prevalence and Lack of Cross-Sectional Association Between Positive Lactulose Breath Test Findings and Fecal Calprotectin Positivity in Patients with Functional Diarrhea

**DOI:** 10.3390/diagnostics16183012

**Published:** 2026-09-17

**Authors:** Susie Jung, Kwang-Min Kim, Kyucheol Lee, Sang-Hoon Lee, Soohwan Jung, Kyu-Nam Kim, Jiyoung Lee

**Affiliations:** 1 Department of Family Practice and Community Health, School of Medicine, Ajou University, Suwon 16499, Republic of Korea; lovehrh@naver.com (S.J.); gaksi@ajou.ac.kr (K.-M.K.); 2 Cheongdamrui Plastic Surgery, Jeju-si 63080, Republic of Korea; cdruilee@gmail.com; 3 Functional Medicine Clinic, Greencross I-MED Gangnam Center, Seoul 06627, Republic of Korea; shlee0612@naver.com; 4 Philip Medical Center, Seongnam-si 13590, Republic of Korea; 2027fmlove@naver.com; 5 Department of Biochemistry & Molecular Medicine, GW Cancer Center, School of Medicine & Health Sciences, The George Washington University, Washington, DC 20037, USA

**Keywords:** functional diarrhea, small intestinal bacterial overgrowth, intestinal methanogen overgrowth, fecal calprotectin

## Abstract

**Background/Objectives:** Functional diarrhea (FDr) is a common gastrointestinal disorder, and alterations in the gut microbiota have been proposed as one of its potential pathophysiological mechanisms. Hydrogen/methane breath tests are commonly used to identify findings suggestive of small intestinal bacterial overgrowth (SIBO) and intestinal methanogen overgrowth (IMO), whereas fecal calprotectin (FC) serves as a marker of intestinal inflammation. However, the prevalence of these findings and their relationship in patients with FDr remain unclear. This study aimed to determine the prevalence of positive breath test findings (suggestive of SIBO/IMO) and detectable/elevated FC in patients with a diagnosis of FDr and to evaluate their cross-sectional relationship and phenotypic overlap. **Methods:** We retrospectively reviewed patients who underwent both a lactulose breath test and FC measurement between March 2015 and April 2021 and fulfilled the Rome IV criteria for FDr. Positive breath test findings suggestive of SIBO/IMO were defined as a hydrogen increase of ≥20 ppm within 90 min or a methane increase of ≥10 ppm at any time following ingestion of 10 g of lactulose. FC was evaluated at two thresholds: the analytical limit of detection (LOD ≥ 11.5 mg/kg), used as an exploratory threshold to distinguish detectable from non-detectable FC, and the conventional clinical cutoff (>50 mg/kg), used to identify clinically elevated FC levels. **Results:** Among 524 patients with FDr aged ≥ 20 years, 176 (33.6%) had positive breath test findings suggestive of SIBO/IMO, 177 (33.8%) had detectable FC (≥11.5 mg/kg), and 71 (13.5%) met the conventional cutoff (>50 mg/kg). Concurrent breath test positivity and detectable FC (≥11.5 mg/kg) were present in 52 patients (9.9%), while only 19 patients (3.6%) exhibited concurrent breath test positivity and clinically elevated FC (>50 mg/kg). **Conclusions:** Approximately one-third of patients with Rome IV-defined FDr demonstrated positive breath test findings suggestive of SIBO/IMO, and a similar proportion had detectable FC concentrations (≥11.5 mg/kg), whereas clinically elevated FC (>50 mg/kg) was present in 13.5% (71/524). Positive breath test findings and FC elevation showed no significant cross-sectional association at either threshold. These findings provide descriptive prevalence estimates and do not establish mechanistic relationships between breath test positivity and FC elevation in patients with Rome IV-defined FDr.

## 1. Introduction

Functional diarrhea (FDr) is a common gastrointestinal disorder, affecting nearly 5% of the global population, and is a frequent cause of clinical visits [1,2]. Based on the Rome IV criteria, FDr is characterized by persistent loose or watery stools in more than 25% of bowel movements in the absence of an identifiable organic cause and without predominant abdominal pain [3]. FDr can lead to a marked deterioration in quality of life and is commonly associated with symptoms such as persistent fatigue, headache, and dizziness. Therefore, identifying its underlying pathophysiology is vital for effective management [4,5]. However, the precise etiology of FDr continues to be elusive, and management is typically supportive. Symptoms frequently recur after cessation of therapy, indicating unresolved underlying mechanisms.

Recent studies suggest that alterations in the gut microbial ecosystem may be associated with the clinical manifestations of FDr [6,7,8]. Furthermore, interventions aimed at correcting gut dysbiosis have demonstrated symptom relief, with these benefits sometimes continuing beyond the cessation of antibiotics such as rifaximin—a non-absorbable agent used for dysbiosis treatment [9,10]. Clinically, gut dysbiosis may affect both the small and large intestines. Because these anatomical regions harbor distinct microbial ecosystems and exhibit different physiological functions, evaluating microbial alterations requires region-specific clinical markers.

In the small intestine, dysbiosis commonly manifests as an abnormal overgrowth of bacteria or methanogenic archaea, termed small intestinal bacterial overgrowth (SIBO) or intestinal methanogen overgrowth (IMO), respectively [11]. While endoscopic sampling and culturing of small intestinal contents are available, these procedures are invasive and do not consistently yield reliable results. As a result, breath testing using carbohydrate substrates has become the primary noninvasive diagnostic tool to identify findings suggestive of SIBO and IMO in clinical practice.

In addition to small intestinal alterations, evaluating pathophysiological changes in the lower gastrointestinal tract is crucial for a comprehensive understanding of FDr. However, routine monitoring of the lower bowel remains challenging due to the invasiveness of mucosal tissue sampling. Fecal calprotectin (FC) has emerged as a reliable, noninvasive surrogate marker of neutrophil-mediated intestinal inflammation [12].

Accumulating clinical evidence indicates that targeting SIBO/IMO alleviates diarrheal symptoms [9,13], while separate interventions modulating colonic dysbiosis have demonstrated concurrent symptom resolution and reductions in FC levels [7,8]. Although both small intestinal dysbiosis (reflected by breath test positivity) and colonic mucosal inflammation (reflected by elevated FC) have been separately implicated in functional bowel disorders, they have predominantly been investigated in isolation or primarily within irritable bowel syndrome (IBS). Consequently, it remains unclear whether positive breath test findings and FC elevation tend to co-occur or are cross-sectionally associated in patients strictly diagnosed with Rome IV FDr. Establishing the co-occurrence and cross-sectional relationship between these two noninvasive biomarkers directly addresses this critical knowledge gap. Therefore, this study aimed to determine the prevalence of breath test positivity and detectable/elevated FC and to evaluate their cross-sectional relationship and degree of overlap in a large cohort of patients with FDr.

## 2. Materials and Methods

### 2.1. Study Design

This retrospective study was conducted at Ajou University Hospital. Patient records from March 2015 to April 2021 were reviewed to identify individuals who had presented to the Department of Family Medicine and Community Health with gastrointestinal symptoms and had undergone both a lactulose breath test and FC measurement. To reduce potential confounding due to other gastrointestinal diseases, such as peptic ulcer disease, gastric cancer, inflammatory bowel disease, or colorectal cancer, we also analyzed endoscopic reports, specifically gastroscopy performed within the previous two years and colonoscopy conducted within the preceding three years.

**Figure 1:**
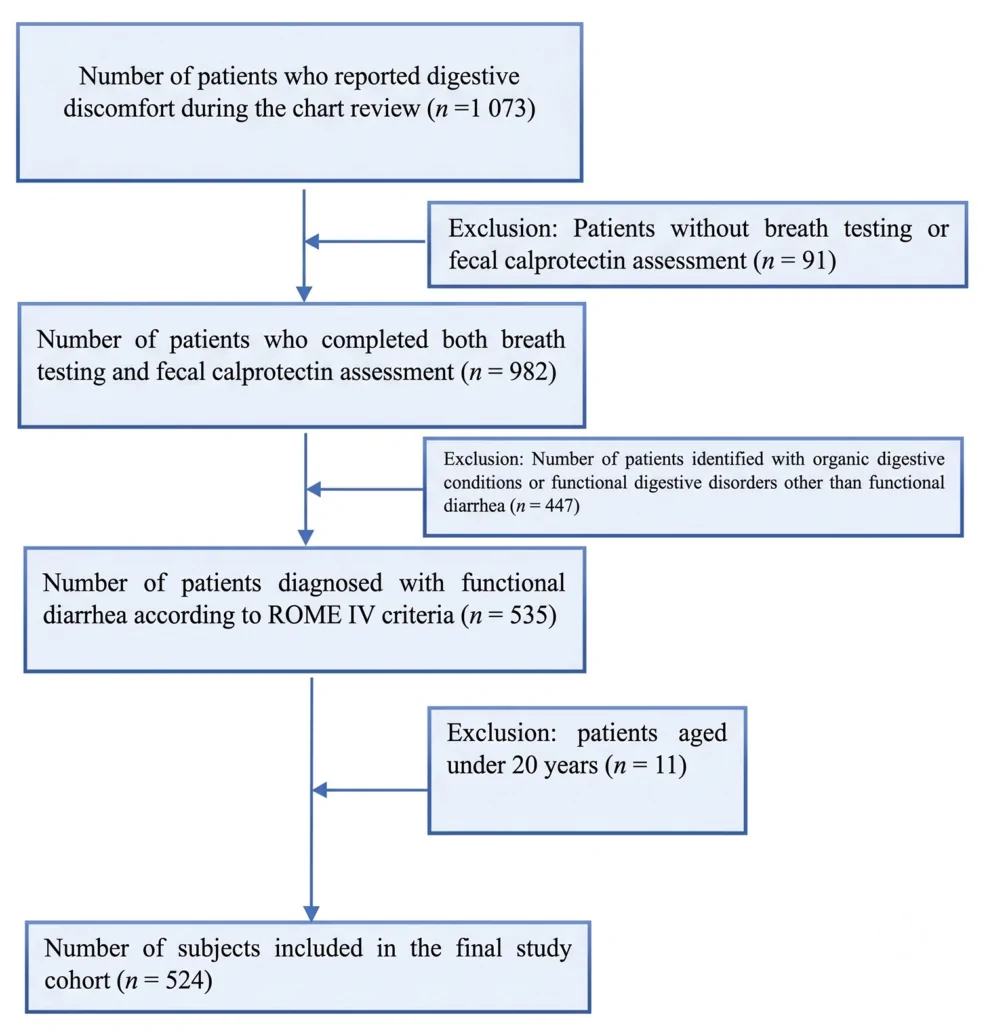
Flow diagram representing the structure of the current study.

We initially screened 1073 patients presenting with gastrointestinal symptoms, of whom 91 did not complete both tests, leaving 982 eligible patients. Study inclusion criteria were based on the Rome IV definition for FDr, requiring loose or watery stools (Bristol Stool Form Scale type 6 or 7) in more than 25% of bowel movements for at least 3 months, with symptom onset at least 6 months prior to diagnosis. In strict adherence to Rome IV guidelines, patients fulfilling the criteria for IBS with diarrhea (IBS-D)—defined by recurrent abdominal pain occurring at least 1 day per week associated with defecation or a change in stool frequency/form—were excluded. Furthermore, other functional gastrointestinal disorders (e.g., functional constipation) and organic gastrointestinal diseases were systematically excluded through endoscopic and clinical review (n = 447), yielding 535 patients who met the Rome IV criteria for FDr. Non-pain abdominal symptoms, such as mild-to-moderate bloating or distension, did not constitute exclusion criteria. Following the exclusion of 11 patients aged under 20 years, a final cohort of 524 patients was included in the statistical analysis (Figure 1).

### 2.2. Data Collection

Demographic data, medical history, alcohol consumption, liver function assessments, bowel movement frequency, stool consistency, and abdominal symptoms were systematically recorded. Comorbidities such as hypertension, dyslipidemia, and diabetes were confirmed either by review of medical records or through direct patient interviews. Weekly alcohol intake was calculated using the quantity–frequency method [14]. For research purposes, the medical records and clinical data were accessed and analyzed between 29 October 2025 and 31 December 2025. During and after data collection, the authors had no access to information that could identify individual participants, as all data were fully anonymized prior to analysis. According to our institutional protocol, breath testing and FC assays were scheduled following a mandatory 4-week cessation of antibiotics, probiotics, prokinetics, and proton pump inhibitors; individuals with documented exposure during this window were systematically identified and excluded upon medical chart review.

### 2.3. FC Measurement

Participants received stool collection kits with instructions to collect and refrigerate samples immediately prior to their visit and submit them on the day of the breath test. Thus, stool sample collection and breath testing were scheduled concurrently on the same clinic visit day (with stool collected within 24 h prior to the breath test) following the 4-week washout of antibiotics, probiotics, prokinetics, and proton pump inhibitors, ensuring direct temporal comparability between both biomarker assessments. FC concentrations were measured in mg/kg using a fluorescence enzyme immunoassay (FEIA) kit (Phadia GmbH, Freiburg, Germany) on an ImmunoCAP 250 analyzer (measuring range: 11.5–3000 mg/kg; intra- and inter-assay coefficients of variation < 10%) at Green Cross LabCell, Yongin, Korea. Given that FDr is generally characterized by functional disturbances rather than overt mucosal inflammation, FC concentrations were evaluated using two thresholds: (1) the analytical limit of detection (LOD ≥11.5 mg/kg), used as an exploratory threshold to distinguish detectable from non-detectable FC [7,8,15], and (2) the conventional clinical cutoff of >50 mg/kg, used to identify clinically elevated FC levels [16]. Values below the analytical LOD (<11.5 mg/kg) were treated as non-detectable for categorical comparisons, tied-ranked for continuous non-parametric tests, and imputed at the LOD threshold (11.5 mg/kg) solely for scatter plot logarithmic visualization.

### 2.4. Breath Testing

Lactulose hydrogen/methane breath tests were conducted after a 12 h fasting period using a BreathTracker SC Quintron gas chromatograph (Quintron Instrument Company, Milwaukee, WI, USA). After baseline sampling, participants ingested 10 g of lactulose dissolved in 15 mL of water, according to the institutional protocol routinely used in our center and previous studies [9]. Breath samples were collected every 20 min during the first hour and every 15 min during the second hour to capture both early and later hydrogen/methane responses over the 120 min testing period. According to our institutional protocol, pre-test quality control required a baseline fasting hydrogen level <16 ppm; patients exceeding this threshold were questioned regarding dietary compliance and rescheduled. In accordance with the North American Consensus guidelines [17], breath test positivity suggestive of SIBO was defined as an increase in hydrogen concentration of ≥ 20 ppm from baseline within 90 min. Breath test positivity suggestive of IMO was defined as a methane concentration of ≥10 ppm at any time during the test (including baseline, reflecting generalized methanogenic colonization) [17].

### 2.5. Statistical Analysis

All statistical analyses were conducted using IBM SPSS Statistics version 24 (IBM Corp., Armonk, NY, USA). Continuous variables were expressed as mean ± standard deviation (SD) or as median with interquartile range (IQR, 25th–75th percentiles) depending on data distribution. Data normality was evaluated using the Shapiro–Wilk test. FC concentrations exhibited a highly skewed distribution and were compared between breath test-positive and breath test-negative groups using the non-parametric Mann–Whitney U test. Categorical variables were presented as frequencies with percentages and evaluated using Pearson’s chi-square (χ^2^) test (with degrees of freedom specified) or Fisher’s exact test, as appropriate. Prevalence rates with 95% confidence intervals (CIs) for SIBO (H_2_-positive), IMO (CH_4_-positive), any SIBO/IMO positivity, and detectable/clinically elevated FC were computed using the Wilson score method.

To evaluate the quantitative relationship between breath gas production and FC concentrations on a continuous scale, correlations between cumulative gas production over the initial 90 min period (area under the curve for hydrogen [AUC__H2(0–90 min)_] and methane [AUC__CH4(0–90 min)_], ppm·min) and FC concentrations (mg/kg) were assessed using Spearman’s rank correlation coefficient (rS). For visual representation in scatter plots, FC values below the analytical LOD (LOD < 11.5 mg/kg) were imputed as the LOD threshold prior to natural logarithmic transformation (Ln FC).

To evaluate potential residual confounding, multivariable binary logistic regression analyses were performed to assess the adjusted association between breath test positivity and FC status across both thresholds (≥11.5 mg/kg and >50 mg/kg), adjusting for age, sex, body mass index, weekly alcohol consumption, hypertension, diabetes mellitus, and dyslipidemia. Two-tailed *p*-values < 0.05 were considered statistically significant.

### 2.6. Use of Artificial Intelligence Tools

During the preparation of this manuscript, generative artificial intelligence (Gemini, Google) was utilized solely to assist with English language editing, grammatical correction, and enhancement of overall readability. The study design, data collection, statistical analysis, interpretation of data, and scientific conclusions were conducted entirely by the human authors. All AI-assisted suggestions were critically reviewed, edited, and approved by the authors, who take full responsibility for the final contents of this publication.

### 2.7. Ethics Statement

The study protocol was approved by the Institutional Review Board of Ajou University Hospital (Approval No. AJOUIRB-DB-2025-159). The requirement for informed consent was waived due to the retrospective nature of the study and the use of anonymized clinical data.

## 3. Results

Table 1 details the baseline characteristics of the study participants. The analysis included 524 patients diagnosed with FDr. The mean age was 48.4 ± 11.3 years. Of these, 349 were male, representing 66.6% of the sample. The average body mass index was 24.7 ± 3.6 kg/m^2^. For lifestyle habits, mean weekly alcohol intake was 61 ± 44.5 g, while 23.7% of patients reported current smoking. Regarding laboratory parameters, serum alanine aminotransferase averaged 32.1 ± 18.5 IU/L, aspartate aminotransferase averaged 26.2 ± 13.4 IU/L, and gamma-glutamyl transferase averaged 37 ± 38.2 IU/L. For concomitant health conditions, 9.5% were on antihypertensive therapy, 6.6% were receiving antidiabetic medications, and 7.0% were prescribed lipid-lowering agents.

To evaluate potential imbalances and confounding factors, general clinical characteristics were sequentially compared between stratified groups. First, demographic and clinical features were evaluated by comparing patients with breath test positivity (n = 176) against those with negative results (n = 348) (Table 2). There were no statistically significant differences between the breath test-positive and breath test-negative groups with respect to age (49.1 ± 12.4 vs. 47.8 ± 10.7 years; *p* = 0.293), male predominance (68.2% vs. 65.8%; *p* = 0.586), or body mass index (24.5 ± 3.7 vs. 25.1± 3.7 kg/m^2^; *p* = 0.082). Furthermore, lifestyle factors such as alcohol consumption (60.1 ± 42.6 vs. 62.7 ± 45.6 g/week; *p* = 0.525) and the prevalence of metabolic comorbidities—including hypertension (8.5% vs. 10.1%; *p* = 0.573), diabetes mellitus (7.4% vs. 6.3%; *p* = 0.482), and dyslipidemia (7.4% vs. 6.9%; *p* = 0.596)—were comparably distributed between the two groups.

Subsequently, general characteristics were compared between patients with detectable FC (n = 177) and those with undetectable FC (n = 347) to assess factors associated with FC status (Table 3). Age, sex, alcohol consumption, hypertension, diabetes mellitus, and dyslipidemia did not differ significantly between the two groups. Body mass index was modestly higher in patients with detectable FC than in those with undetectable FC (25.1 ± 3.9 vs. 24.4 ± 3.6 kg/m^2^; *p* = 0.047).

Table 4 presents the prevalence of SIBO, IMO, any SIBO/IMO positivity, and detectable or clinically elevated FC levels in patients with FDr. Among the 524 patients, the prevalence of SIBO (H_2_-positive) was 18.9% (99/524; 95% CI, 15.8–22.5%), IMO (CH_4_-positive) was 21.4% (112/524; 95% CI, 18.1–25.1%), and any SIBO/IMO positivity was 33.6% (176/524; 95% CI, 29.7–37.7%). For FC, detectable FC (≥11.5 mg/kg) was observed in 177 patients (33.8%; 95% CI, 29.9–37.9%), whereas clinically elevated FC (>50 mg/kg) was identified in 71 patients (13.5%; 95% CI, 10.8–16.8%).

**Figure 2:**
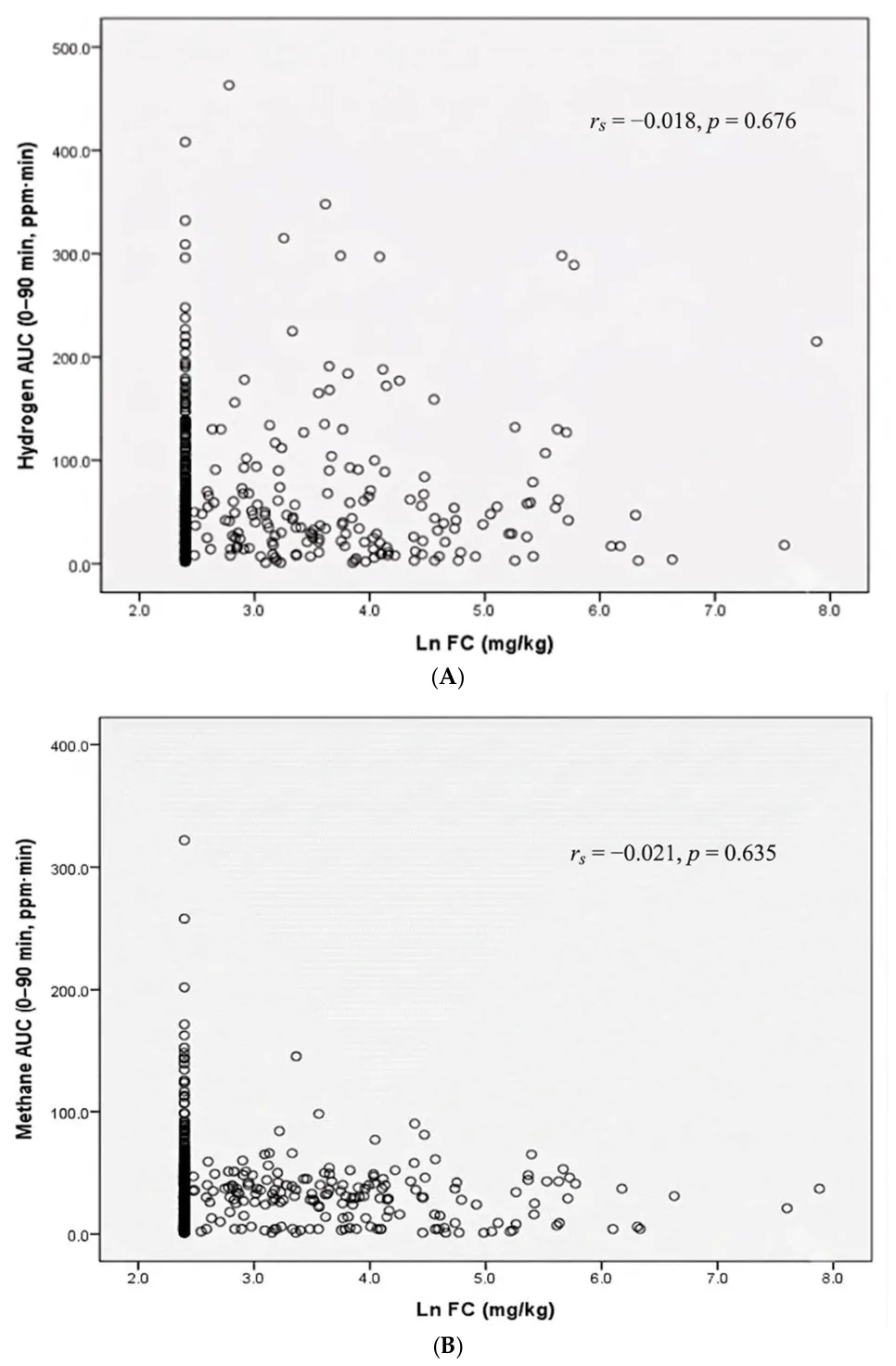
Correlations between cumulative breath gas excretion and fecal calprotectin (FC) levels in patients with functional diarrhea (n = 524). (**A**) Hydrogen cumulative excretion over 90 min (AUC_0–90min_) plotted against natural log-transformed fecal calprotectin (Ln FC) (*r_S_* = −0.018, *p* = 0.676). (**B**) Methane cumulative excretion over 90 min (AUC_0–90min_) plotted against Ln FC (*r_S_* = −0.021, *p* = 0.635). Values below the analytical limit of detection (FC < 11.5 mg/kg) were imputed as the detection threshold (11.5 mg/kg, corresponding to Ln [11.5] ≈ 2.44) prior to logarithmic transformation. Spearman’s rank correlation analysis evaluated ordinal ranks of raw values; logarithmic transformation was applied solely for scatter plot visualization. AUC, area under the curve; FC, fecal calprotectin.

Cross-tabulation analysis demonstrating the phenotypic overlap between SIBO/IMO positivity and FC status across both thresholds is summarized in Table 5. At the LOD threshold (≥11.5 mg/kg), 52 patients (9.9%) were concurrently positive on breath testing and for detectable FC, 124 (23.7%) were positive for SIBO/IMO alone, and 125 (23.9%) were positive for detectable FC alone, demonstrating no statistically significant association (odds ratio [OR] = 0.75, 95% CI: 0.51–1.11). Similarly, when applying the conventional cutoff (>50 mg/kg), concurrent positivity was observed in only 19 patients (3.6%), with no significant association (OR = 0.69, 95% CI: 0.40–1.20). To determine whether baseline demographic, lifestyle, or metabolic factors confounded these observations, multivariable logistic regression analyses were conducted adjusting for age, sex, body mass index, weekly alcohol intake, hypertension, diabetes mellitus, and dyslipidemia. After multivariable adjustment, breath test positivity remained not significantly associated with detectable FC at the LOD threshold (≥11.5 mg/kg; adjusted odds ratio [aOR] = 0.82, 95% CI: 0.48–1.38, *p* = 0.450) or with clinically elevated FC at the conventional threshold (>50 mg/kg; aOR = 0.82, 95% CI: 0.40–1.68, *p* = 0.583). In addition to categorical cross-tabulations, we investigated whether quantitative breath gas production correlated with FC concentrations across the entire cohort (n = 524). Spearman’s rank correlation analysis revealed no statistically significant correlation between cumulative hydrogen excretion (AUC_H2(0–90min)_) and FC levels (*r_S_* = −0.018, *p* = 0.676) or between cumulative methane excretion (AUC_CH4(0–90min)_) and FC levels (*r_S_* = −0.021, *p* = 0.635) (Figure 2).

Across the entire cohort (n = 524), FC concentrations showed a marked right-skewed distribution, ranging from <11.5 to 2647.0 mg/kg with a mean of 42.4 ± 159.1 mg/kg. The overall median FC level was <11.5 mg/kg (IQR: <11.5–22.7 mg/kg). When evaluated on a continuous scale, FC concentrations did not differ significantly between breath test-positive patients (n = 176; median: <11.5 mg/kg, IQR: <11.5–22.7 mg/kg; mean rank: 249.92) and breath test-negative patients (n = 348; median: <11.5 mg/kg, IQR: <11.5–22.7 mg/kg; mean rank: 268.86; Mann–Whitney U = 28,409.5, Z = −1.606, *p* = 0.108).

**Figure 3:**
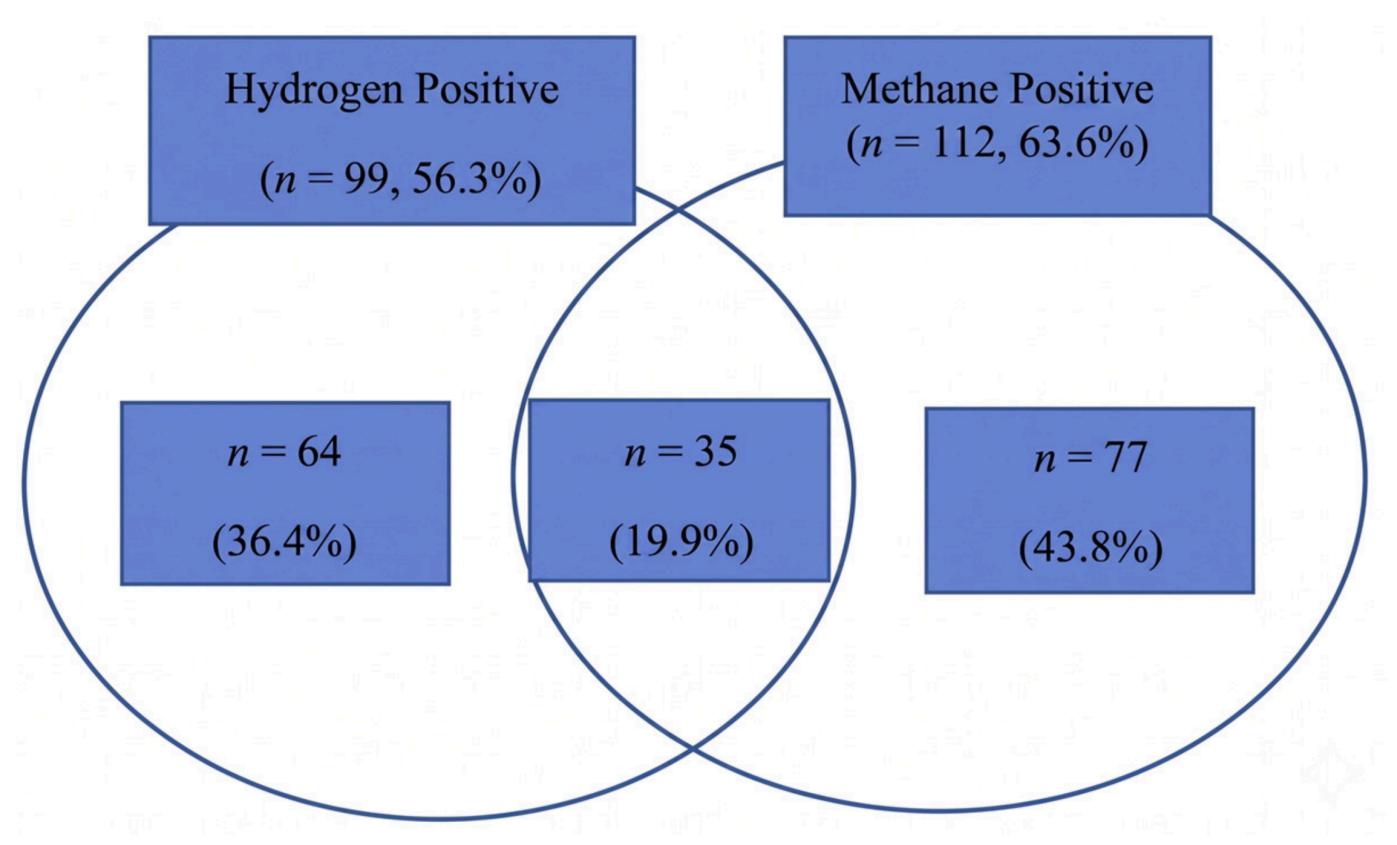
Distribution of breath test positivity subtypes among all hydrogen/methane-positive individuals (n = 176). Among these, 112 patients (63.6%) were methane-positive, and 99 (56.3%) were hydrogen-positive. Proportions relative to the total hydrogen/methane-positive group (n = 176) were: methane-positive only, 43.8% (n = 77; representing 68.8% of the methane-positive group [77/112]); concurrent methane- and hydrogen-positive, 19.9% (n = 35; representing 31.3% of the methane-positive group [35/112] and 35.4% of hydrogen-positive patients [35/99]); and hydrogen-positive only, 36.4% (n = 64; representing 64.6% of the hydrogen-positive group [64/99]).

The proportions of hydrogen- and methane-producing subtypes within the breath test-positive group are displayed in Figure 3. Within the subgroup with positive breath test findings (n = 176), IMO positivity was more prevalent than SIBO positivity (63.6% [112/176] vs. 56.3% [99/176]). Among all 176 SIBO/IMO-positive individuals, 43.8% (77/176; representing 68.8% of all IMO-positive patients [77/112]) exhibited IMO alone, 19.9% (35/176; representing 31.3% of IMO-positive [35/112] and 35.4% of SIBO-positive patients [35/99]) were positive for both IMO and SIBO, and 36.4% (64/176; representing 64.6% of all SIBO-positive patients [64/99]) were classified as having SIBO alone.

To address potential differences between hydrogen- and methane-producing subtypes, FC positivity was evaluated separately across SIBO-alone (n = 64), IMO-alone (n = 77), dual-positive (n = 35), and breath test-negative (n = 348) groups (Table 6). At the analytical LOD threshold (≥11.5 mg/kg), detectable FC was observed in 39.1% of SIBO-alone, 23.4% of IMO-alone, 25.7% of dual-positive, and 35.9% of breath test-negative patients (*p* = 0.100 across all four groups; *p* = 0.109 among the three positive subtypes). Similarly, using the conventional cutoff (>50 mg/kg), clinically elevated FC was present in 15.6% of SIBO-alone, 9.1% of IMO-alone, 5.7% of dual-positive, and 14.9% of breath test-negative patients (*p* = 0.262 across all four groups; Fisher’s exact *p* = 0.279 among the three positive subtypes). Although SIBO-alone exhibited numerically higher rates of FC positivity than methanogenic subtypes, no statistically significant association between specific gas subtypes and mucosal inflammatory status was identified.

## 4. Discussion

In this study, the prevalence of positive breath test findings suggestive of SIBO/IMO and FC elevation was assessed in patients with FDr. Approximately one-third of the cohort had either positive breath test findings or detectable FC concentrations (≥11.5 mg/kg), whereas only 9.9% had both findings. However, no statistically significant cross-sectional association was observed between positive breath test findings and FC elevation. As our data are observational, these findings should be interpreted as descriptive evidence of limited overlap rather than as evidence of biological independence.

While prior studies have detailed the prevalence of SIBO in other functional gastrointestinal disorders such as functional dyspepsia and IBS [18,19,20], this report is, to our knowledge, the first to investigate both SIBO/IMO and elevated FC within a Rome IV-defined cohort of FDr patients.

Meta-analyses indicate that SIBO prevalence ranges from 35% to over 50% in functional dyspepsia, depending on the diagnostic approach [21], and from 31% to 36.7% in IBS, with particularly high rates in IBS-D based on Rome III [20]. Because the Rome IV criteria for FDr are closely aligned with the Rome III criteria for IBS-D, our finding of a breath test positivity rate near 33% aligns with these previous reports [20].

Several pathophysiological mechanisms have been proposed to explain how increased hydrogen production may contribute to diarrheal symptoms. Hydrogen has been shown to stimulate small intestinal motility [22], partly by modulating enterochromaffin serotonin release and peristaltic activity [23], thereby promoting rapid transit and looser stools. Additionally, excessive luminal fermentation generates short-chain fatty acids and osmotic byproducts that promote fluid retention, serving as a driver of osmotic diarrhea and potential subclinical mucosal alterations [24,25]. However, because gut motility and mucosal tissue were not directly evaluated in the present study, these mechanisms represent hypothetical considerations rather than demonstrated findings in our cohort.

In our SIBO/IMO-positive subgroup, methane positivity was observed slightly more frequently than hydrogen positivity (63.6% vs. 56.3%). Although methanogenesis is classically linked to constipation due to the neuromodulatory, transit-slowing effects of methane gas [26], substantial IMO prevalence was observed in this FDr cohort. This apparent paradox can be explained by several mechanisms. First, co-production of hydrogen and short-chain fatty acids may exert predominant osmotic and prokinetic drives that override the transit-slowing effect of methane [24,27]. Second, methanogens such as *Methanobrevibacter smithii* may colonize the colonic or distal small bowel lumen and generate detectable methane gas without inducing sufficient contractile inhibition to cause overt constipation [28]. Furthermore, host-specific variations in mucosal immune activation and visceral sensitivity may alter clinical manifestations [29,30], suggesting that breath methane serves as a marker of methanogenic colonization rather than an absolute predictor of slowed transit.

FC is a well-established marker of neutrophil-mediated intestinal inflammation and has been widely used in the evaluation of inflammatory bowel disease (IBD) [16,31]. In the present study, elevated FC levels were identified in approximately one-third of patients with FDr when using the analytical detection threshold. However, as this lower threshold represents the analytical LOD rather than an established clinical cutoff for active mucosal inflammation, detectable values may partly reflect background assay noise. Indeed, when examined as a continuous variable, the median FC level in the cohort was below the analytical LOD (<11.5 mg/kg), and continuous concentrations showed no statistically significant difference between breath test-positive and breath test-negative groups. The clinical significance of low-level FC elevations in functional gastrointestinal disorders remains uncertain. Previous studies have suggested that subtle increases in FC may be associated with low-grade inflammatory activity or alterations in gut microbial composition, but definitive evidence remains limited [12,32]. Given the lack of a direct baseline correlation with breath gas profiles in our study, minor FC elevations should be interpreted cautiously as a nonspecific sign of intestinal tissue response rather than as a surrogate marker for colonic dysbiosis. Therefore, further studies are required to clarify the biological and clinical implications of FC elevation in patients with FDr.

Notably, the absence of an association remained consistent regardless of whether the sensitive LOD cutoff (≥11.5 mg/kg) or the conventional inflammatory threshold (>50 mg/kg) was applied. These concordant results indicate an absence of a significant cross-sectional association between positive breath test findings and FC elevation across both cutoffs. This lack of measurable overlap across the cohort was observed despite broadly similar distributions of major clinical and demographic variables. Although body mass index differed modestly between the detectable-FC and undetectable-FC groups, the multivariable analysis adjusted for body mass index as well as age, sex, weekly alcohol intake, hypertension, diabetes, and dyslipidemia, and the absence of a significant association between breath test positivity and FC status remained unchanged. While these findings show that positive breath test findings and elevated FC exhibited limited cross-sectional overlap in individual patients, they should be interpreted cautiously as descriptive observations rather than definitive proof of distinct biological mechanisms.

In addition, previous studies conducted by our group in patients with Rome III-defined IBS-D demonstrated that rifaximin treatment was associated with symptomatic improvement accompanied by normalization of breath test findings and reductions in FC levels [9,15]. Although the present study did not evaluate treatment outcomes, these observations suggest that the abnormalities identified in this study may have potential clinical relevance and warrant further prospective investigation.

From a diagnostic perspective, the lack of a cross-sectional association and limited overlap between positive breath test findings and elevated FC highlight the heterogeneity of pathophysiological profiles in FDr. Because positive breath test findings and detectable FC concentrations showed limited cross-sectional overlap without a significant association, a single noninvasive biomarker cannot capture the spectrum of gastrointestinal alterations in this population. Rather than directly supporting targeted therapies—such as gut-directed antimicrobials for overgrowth [9,10] or mucosal-protective agents for inflammatory subsets [7,8]—our findings provide descriptive baseline evidence that proximal fermentative gas production and FC elevation were not significantly associated in this cross-sectional cohort. Future prospective studies evaluating longitudinal treatment responses and symptom outcomes are needed to determine whether these biomarker findings have meaningful clinical utility.

There are several limitations to this study. First, lactulose was used as the substrate for breath testing. In patients with chronic diarrhea, accelerated small bowel transit is frequent and can deliver the non-absorbable substrate to the cecum within 90 min. Consequently, early hydrogen peaks may, in some cases, reflect rapid transit and early colonic fermentation rather than true proximal small intestinal overgrowth, potentially inflating SIBO prevalence estimates. Second, because no healthy control group or comparator population was included, the observed prevalence estimates cannot be considered specific to FDr. Third, a universally accepted FC threshold for functional gastrointestinal disorders has not been established. Therefore, we adopted our laboratory’s analytical LOD (≥11.5 mg/kg) as an exploratory threshold to distinguish detectable from non-detectable FC, consistent with our previous trials [7,8,15]. While applying the conventional cutoff for overt inflammation (>50 mg/kg) yielded a lower prevalence of 13.5% (71/524), detectable FC at the LOD threshold should be interpreted strictly as an analytical observation; in the absence of systematic paired mucosal biopsies or additional tissue inflammatory markers, these values cannot definitively prove low-grade histological inflammation. Fourth, although major organic gastrointestinal diseases were excluded through clinical evaluation and endoscopic review, systematic histologic assessment was not available for all patients, and therefore microscopic causes of chronic diarrhea (such as microscopic colitis) cannot be completely excluded. Finally, because of the retrospective study design, selection bias and residual confounding cannot be completely excluded. While strict 4-week washouts were enforced for antibiotics, probiotics, prokinetics, and proton pump inhibitors, unmeasured factors such as episodic non-steroidal anti-inflammatory drug use or short-term dietary variations were not captured by dietary records, which may have contributed to unmeasured residual variability. Although the comparative groups were broadly similar with respect to major demographic and metabolic variables, body mass index differed modestly between the detectable-FC and undetectable-FC groups and was therefore included in the multivariable analysis.

Despite these limitations, this study is the first to report the prevalence of positive lactulose breath test findings and detectable FC elevations in a relatively large cohort of patients with Rome IV-defined FDr. These findings provide descriptive data that may serve as a basis for future investigations into the clinical and biological significance of these abnormalities.

In conclusion, approximately one-third of patients with Rome IV-defined FDr demonstrated positive lactulose breath test findings suggestive of SIBO/IMO, and a similar proportion exhibited detectable FC, whereas clinically elevated FC was present in 13.5%. The overlap between these findings was limited, and no statistically significant association was observed. These results provide prevalence estimates only and do not establish mechanistic relationships between breath test positivity and FC elevation. Future prospective studies incorporating appropriate control groups, microbiome analyses, and validated inflammatory markers are warranted to clarify the clinical significance of these findings.

## Figures and Tables

**Table 1:** Overview of demographic and general clinical characteristics in patients with functional diarrhea (n = 524).

Characteristics	
Age (year)	48.4 ± 11.3
Male (n, %)	349 (66.6)
Body mass index (kg/m^2^)	24.7 ± 3.6
Alcohol consumption (g/week)	61 ± 44.5
Current smoker (%)	23.7
ALT (IU/L)	32.1 ± 18.5
AST (IU/L)	26.2 ± 13.4
GGT (IU/L)	37.0 ± 38.2
Antihypertensive medication (n, %)	50 (9.5)
Antidiabetic medication (n, %)	35 (6.6)
Lipid-lowering medication (n, %)	37 (7.0)

Data are presented as mean ± standard deviation or as frequency (percentage), as appropriate. ALT, alanine aminotransferase; AST, aspartate aminotransferase; GGT, gamma-glutamyl transferase.

**Table 2:** General characteristics of functional diarrhea patients according to breath test positivity.

Characteristics	Breath Test-Positive (n = 176)	Breath Test-Negative (n = 348)	*p*-Value
Age	49.1 ± 12.4	47.8 ± 10.7	0.293
Men (n, %)	120 (68.2)	229 (65.8)	0.586
Body mass index (kg/m^2^)	24.5 ± 3.7	25.1 ± 3.7	0.082
Alcohol consumption (g/week)	60.1 ± 42.6	62.7 ± 45.6	0.525
Hypertension (n, %)	15 (8.5)	35 (10.1)	0.573
Diabetes mellitus (n, %)	13 (7.4)	22 (6.3)	0.482
Dyslipidemia (n, %)	13 (7.4)	24 (6.9)	0.596

**Table 3:** Comparison of general characteristics between patients with and without detectable fecal calprotectin (FC ≥ 11.5 mg/kg).

Characteristics	Detectable FC (n = 177)	Undetectable FC (n = 347)	*p*-Value
Age	48.5 ± 11.0	48.3 ± 11.5	0.788
Men (n, %)	120 (67.8)	229 (66.0)	0.679
Body mass index (kg/m^2^)	25.1 ± 3.9	24.4 ± 3.6	0.047
Alcohol consumption (g/week)	62.2 ± 40.6	60.9 ± 41.6	0.731
Hypertension (n, %)	18 (10.2)	32 (9.2)	0.727
Diabetes mellitus (n, %)	10 (5.6)	25 (7.2)	0.500
Dyslipidemia (n, %)	12 (6.8)	25 (7.2)	0.857

**Table 4:** Prevalence of positive breath test findings (suggestive of SIBO/IMO) and fecal calprotectin (FC) in patients with functional diarrhea (n = 524).

Variable	N	Positive (n)	Prevalence (%)	95% CI (Wilson)
Hydrogen-positive (suggestive of SIBO)	524	99	18.9%	15.8–22.5%
Methane-positive (suggestive of IMO)	524	112	21.4%	18.1–25.1%
Any breath test-positive	524	176	33.6%	29.7–37.7%
Detectable FC (≥11.5 mg/kg)	524	177	33.8%	29.9–37.9%
Clinically elevated FC (>50 mg/kg)	524	71	13.5%	10.8–16.8%

Data are expressed as frequency (percentage). Confidence intervals were estimated using the Wilson method. SIBO, small intestinal bacterial overgrowth; IMO, intestinal methanogen overgrowth; H_2_, hydrogen gas; CH_4_, methane gas.

**Table 5:** Cross-tabulation of breath test positivity by fecal calprotectin (FC) threshold in patients with functional diarrhea (n = 524).

FC Threshold	Group	FC-Positive	FC-Negative	Total	Odds Ratio (95% CI)
Detectable FC (≥11.5 mg/kg, LOD)	Breath test-positive	52 (9.9%)	124 (23.7%)	176 (33.6%)	0.75 (0.51–1.11)
	Breath test-negative	125 (23.9%)	223 (42.6%)	348 (66.4%)	
	Total	177 (33.8%)	347 (66.2%)	524(100.0%)	
Clinically elevated FC (>50 mg/kg)		FC-Positive	FC-Negative	Total	
	Breath test-positive	19 (3.6%)	157 (30.0%)	176 (33.6%)	0.69 (0.40–1.20)
	Breath test-negative	52 (9.9%)	296 (56.5%)	348 (66.4%)	
	Total	71 (13.5%)	453 (86.5%)	524 (100.0%)	

Data are presented as numbers (percentages). Percentages are calculated relative to the total cohort (n = 524). OR, odds ratio; CI, confidence interval; FC, fecal calprotectin; LOD, limit of detection.

**Table 6:** Fecal calprotectin status across breath test positivity subtypes in patients with functional diarrhea (n = 524).

Breath Test Subtype	Total, n (%)	Detectable FC (≥11.5 mg/kg), n (%)	Clinically Elevated FC (>50 mg/kg), n (%)
Breath test-negative	348 (66.4%)	125 (35.9%)	52 (14.9%)
Hydrogen-positive only (suggestive of SIBO)	64 (12.2%)	25 (39.1%)	10 (15.6%)
Methane-positive only (suggestive of IMO)	77 (14.7%)	18 (23.4%)	7 (9.1%)
Dual-positive (H_2_ and CH_4_)	35 (6.7%)	9 (25.7%)	2 (5.7%)
Total	524 (100.0%)	177 (33.8%)	71 (13.5%)
*p*-value (across 4 groups)		*p* = 0.100 ^a^	*p* = 0.262 ^a^
*p*-value (among 3 positive subtypes)		*p* = 0.109 ^b^	*p* = 0.279 ^c^

Data are expressed as numbers (percentages). SIBO, small intestinal bacterial overgrowth; IMO, intestinal methanogen overgrowth; FC, fecal calprotectin. ^a^ Pearson’s chi-square test across all four groups (df = 3); ^b^ Pearson’s chi-square test among the three breath test-positive subtypes (df = 2); ^c^ Fisher’s exact test among the three breath test-positive subtypes.

## Data Availability

All relevant data supporting the findings of this study are included within the article. Further inquiries can be directed to the corresponding authors.
